# Supplementary material for: Optimising genomic approaches for identifying vancomycin-resistant Enterococcus faecium transmission in healthcare settings
Source: Nat Commun. 2022 Jan 26;13:509. doi: 10.1038/s41467-022-28156-4 (PMC8792028; doi:10.1038/s41467-022-28156-4)
Supplement: Supplementary file 1 — Supplementary Information [file 41467_2022_28156_MOESM1_ESM.pdf]

Optimising genomic approaches for identifying vancomycin-resistant *Enterococcus faecium* transmission

Supplementary Information

Supplementary Table 1. Characteristics and infection control practices of hospital sites included in study

Hospital network	Hospital code	Hospital description	No. of inpatient beds[a]	High-risk wards	MDRO screening practices during study period and changes during study
**A**	**A1**	Tertiary referral center, including ICU, solid organ and bone marrow transplant	560	ICU<br>Hematology/BMT and Oncology<br>Renal Transplant<br>Liver Transplant<br>(Spinal ward and respiratory ward added)	ICU, haematology/oncology, renal and liver transplant wards screened on admission and twice weekly for *vanA* VRE<br>Quarterly point-prevalence survey for *vanA* VRE<br>*Change during study:* Added spinal ward and respiratory ward (ventilator support service) to high-risk wards for regular MDRO screening (October 2018)
	**A2**	Subacute hospital, aged care and rehabilitation services	150	None	Quarterly point-prevalence survey for *vanA* VRE
	**A3**	Subacute hospital, rehabilitation services	60	None	Quarterly point-prevalence survey for *vanA* VRE
**B**	**B1**	Tertiary referral center, including ICU and solid organ transplant and specialist pediatric hospital (including neonatal ICU)	640	ICU<br>Renal Transplant	ICU and renal ward screened for *vanA* VRE on admission and weekly<br>MRSA screening before cardiac surgery<br>*Change during study:* Stopped routine screening of renal ward for VRE (June 2018). Network-wide changes in cleaning practices to from microfibre/steam cleaning to bleach cleaning (September 2018)
	**B2**	Tertiary referral center, including ICU, trauma and some aged care & rehabilitation services	573	ICU	ICU patients screened for *vanA* VRE on admission and weekly<br>*Changes during study:* Cleaning protocol changes as above

**C**	**C1**	Tertiary referral center, including ICU, solid organ and bone marrow transplant	571	ICU Haematology/BMT	ICU and haematology ward screened on admission and weekly for *vanA* VRE
	**C2**	Subacute hospital, aged care and rehabilitation services	150	None	None
**D**	**D1**	Specialized cancer care center. Located adjacent to Hospital 3A (ICU patients cared for at 3A before transfer back to hospital 4)	96	Hematology	Haematology ward patients screened on admission and weekly for *vanA* VRE

ICU, intensive care unit; MRGN, multi-resistant Gram negatives (includes ESBL and carbapenem-resistant phenotypes); BMT, bone marrow transplant (allogeneic) [a]Inpatient beds, excludes day cases, hospital-in-the-home and mental health.

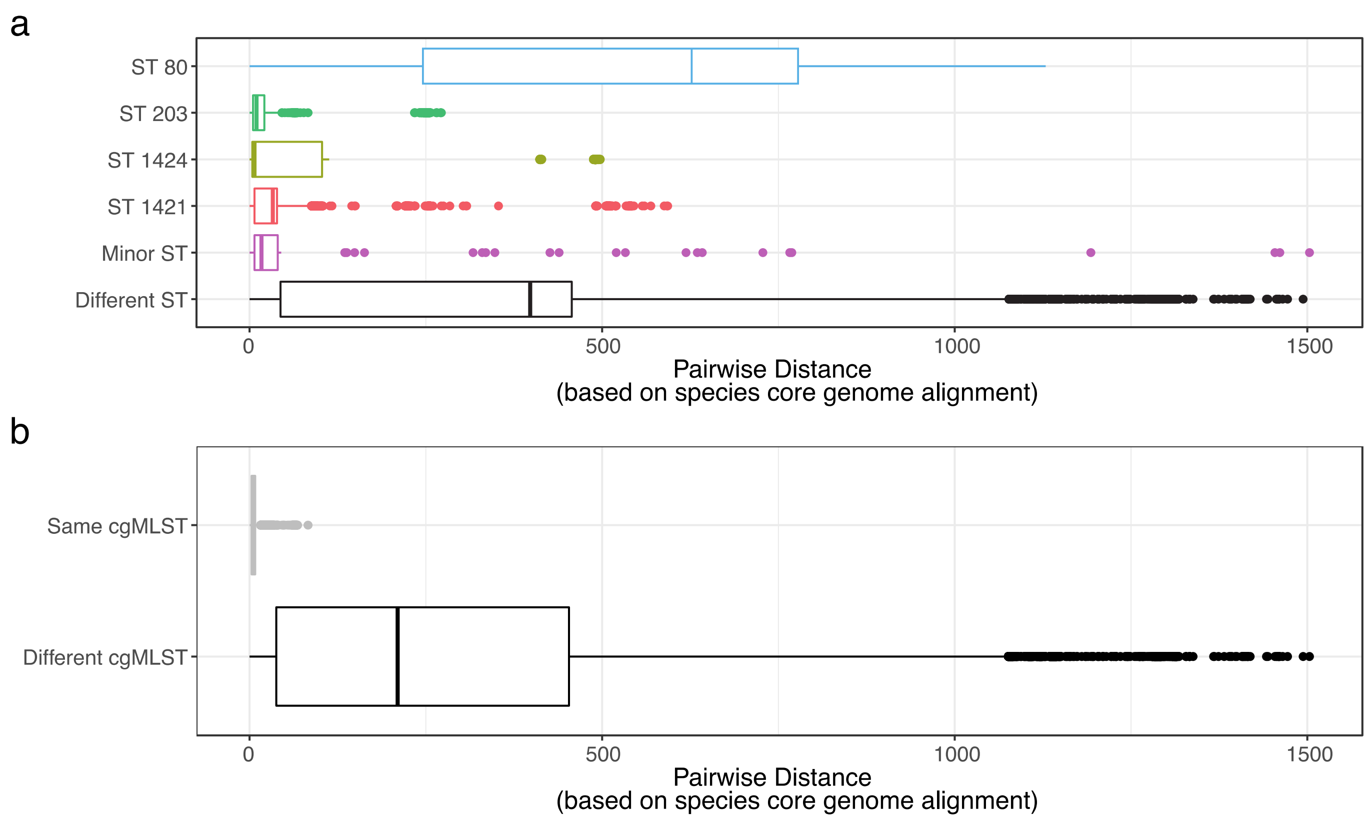

Supplementary Figure 1: Comparison of pairwise SNP distances based on the species level core genome alignment. a) Isolates grouped by ST. b) Isolates grouped by cgMLST cluster. N=343. The lower and upper hinges correspond to the first and third quartiles (the 25th and 75th percentiles) and the line through the box displays the median. The whiskers show 1.5 * IQR with outlying points plotted individually.

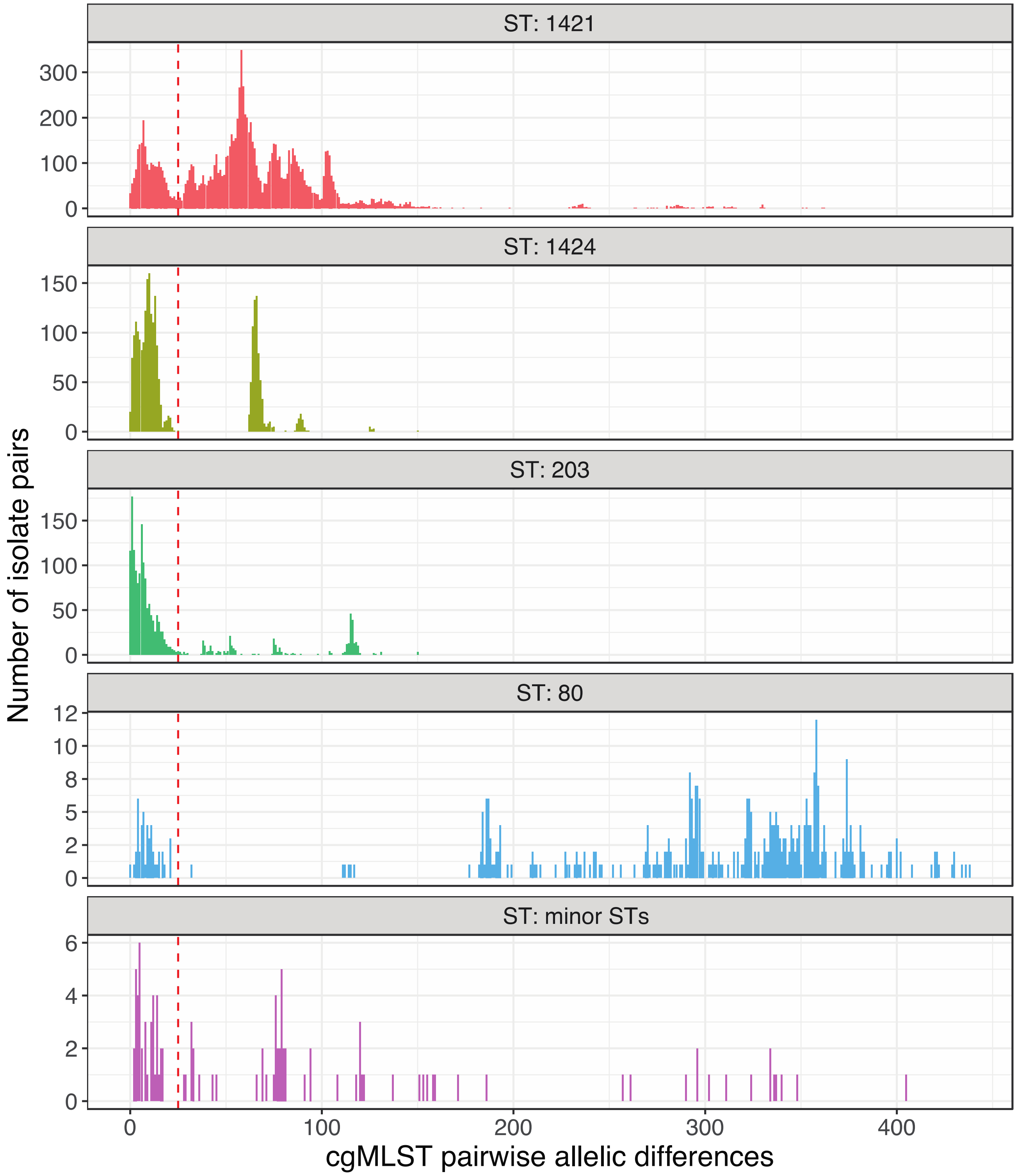

Supplementary Figure 2: Histograms of the cgMLST allelic differences between isolates pairs from within each of the major STs. Histograms are coloured by MLST. The red dashed line represents a pairwise allelic difference threshold of 25. Pairwise allelic differences are shown for within MLST groups only; four each of the four major STs (ST1421, ST1424, ST203 and ST80) and the minor STs.

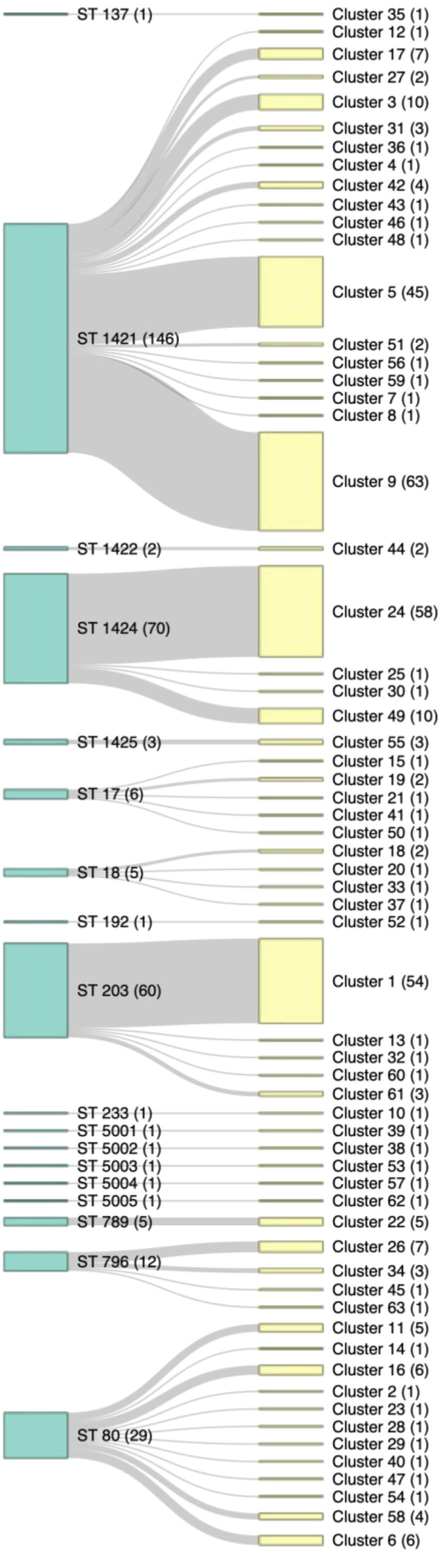

Supplementary Figure 3: Relationship between MLST and cgMLST clusters for all *E. faecium* isolates (n=346). cgMLST clusters are based on a pairwise allelic threshold of less than or equal to 25 and are generated using the single linkage clustering method. The size of the nodes represents the number of isolates in each cluster and the number of isolates in each cluster is displayed in bracket

a

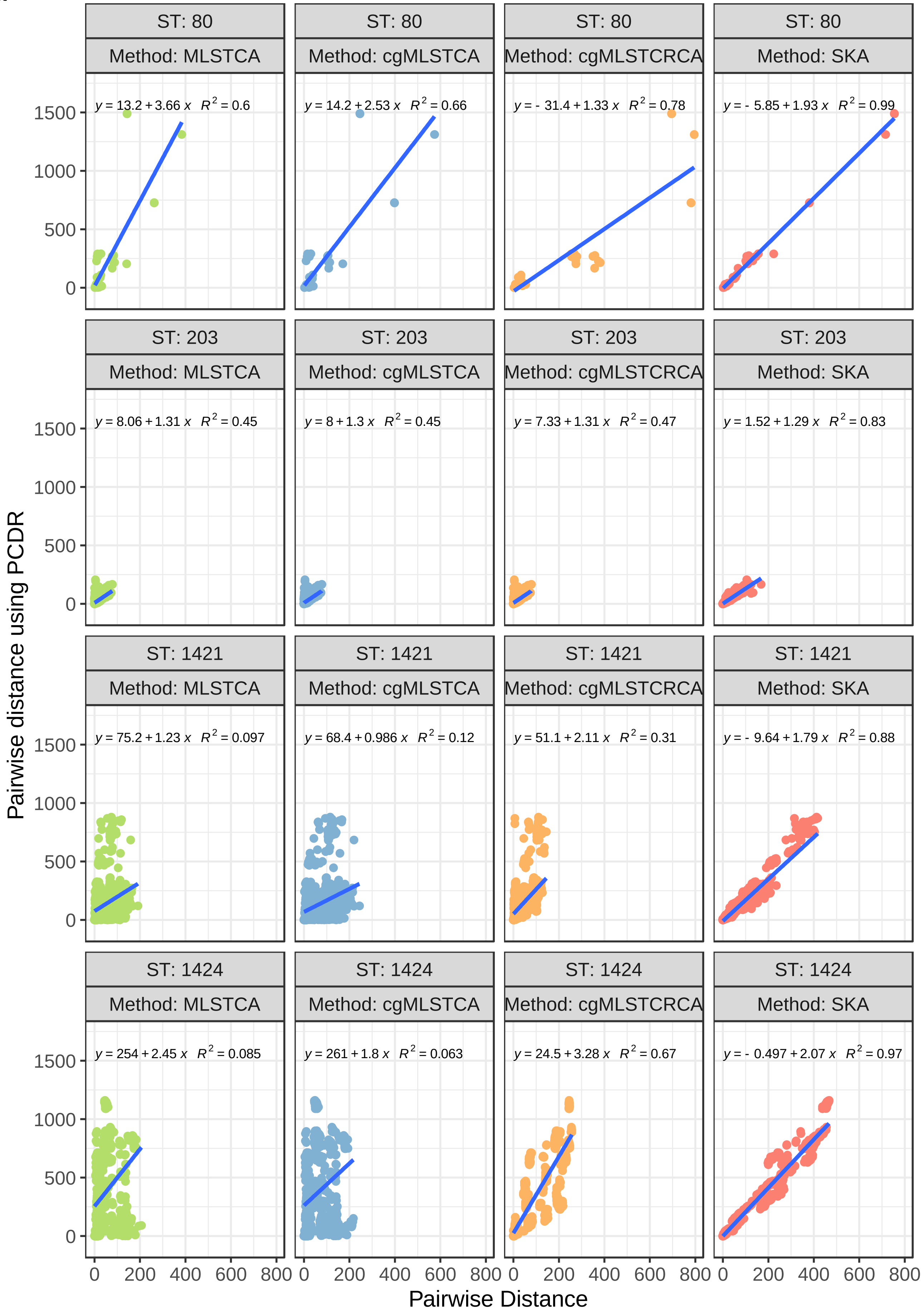

ST: 80
Method: MLSTCA
Method: cgMLSTCA
Method: cgMLSTCRCA
Method: SKA
y = 13.2 + 3.66 x R^2 = 0.6
y = 14.2 + 2.53 x R^2 = 0.66
y = - 31.4 + 1.33 x R^2 = 0.78
y = - 5.85 + 1.93 x R^2 = 0.99
ST: 203
y = 8.06 + 1.31 x R^2 = 0.45
y = 8 + 1.3 x R^2 = 0.45
y = 7.33 + 1.31 x R^2 = 0.47
y = 1.52 + 1.29 x R^2 = 0.83
ST: 1421
y = 75.2 + 1.23 x R^2 = 0.097
y = 68.4 + 0.986 x R^2 = 0.12
y = 51.1 + 2.11 x R^2 = 0.31
y = - 9.64 + 1.79 x R^2 = 0.88
ST: 1424
y = 254 + 2.45 x R^2 = 0.085
y = 261 + 1.8 x R^2 = 0.063
y = 24.5 + 3.28 x R^2 = 0.67
y = - 0.497 + 2.07 x R^2 = 0.97
Pairwise distance using PCDR
Pairwise Distance
0
500
1000
1500
200
400
600
800

b

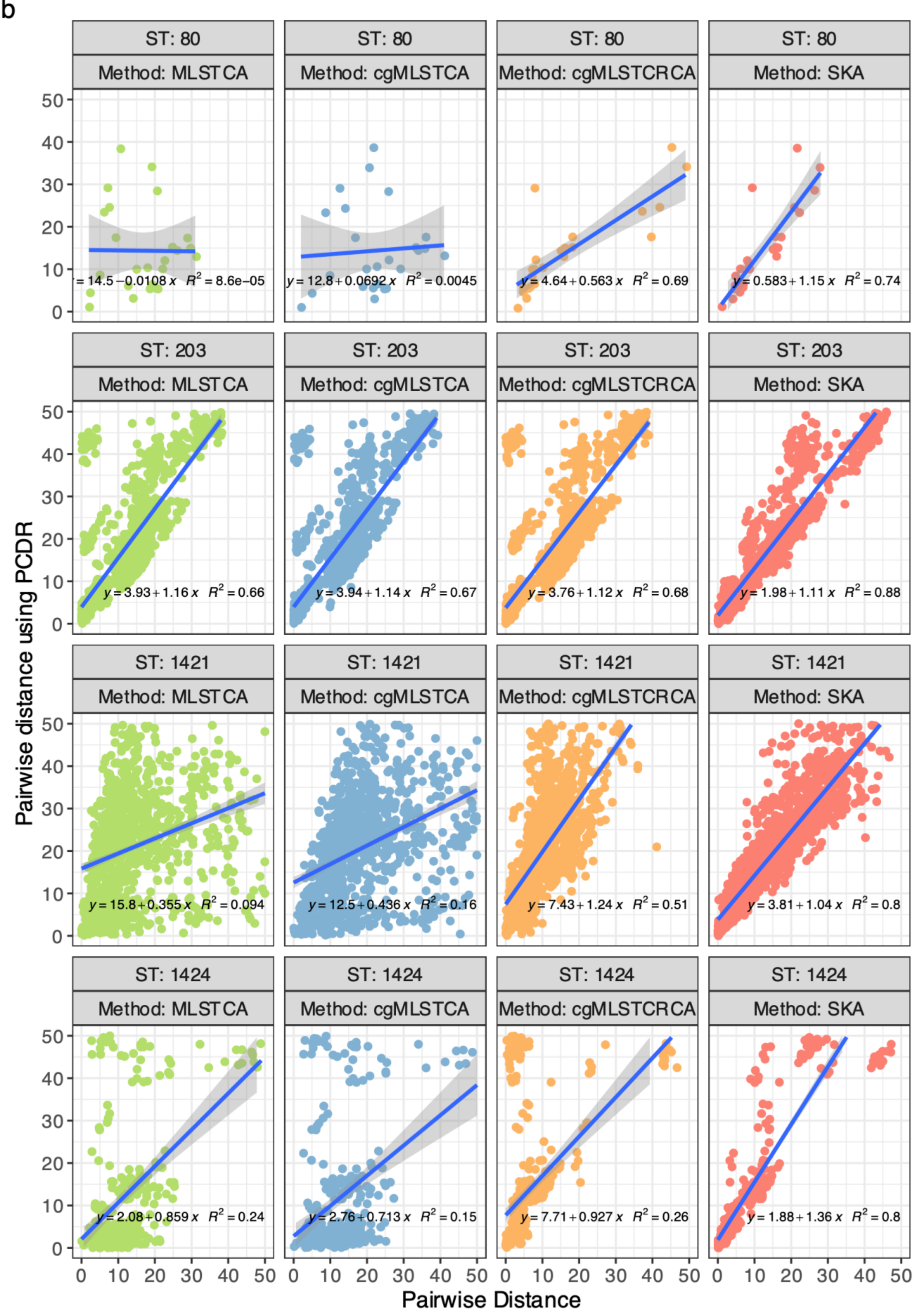

ST: 80
Method: MLSTCA
= 14.5 − 0.0108 x R² = 8.6e−05
ST: 80
Method: cgMLSTCA
y = 12.8 + 0.0692 x R² = 0.0045
ST: 80
Method: cgMLSTCRCA
y = 4.64 + 0.563 x R² = 0.69
ST: 80
Method: SKA
y = 0.583 + 1.15 x R² = 0.74
ST: 203
Method: MLSTCA
y = 3.93 + 1.16 x R² = 0.66
ST: 203
Method: cgMLSTCA
y = 3.94 + 1.14 x R² = 0.67
ST: 203
Method: cgMLSTCRCA
y = 3.76 + 1.12 x R² = 0.68
ST: 203
Method: SKA
y = 1.98 + 1.11 x R² = 0.88
ST: 1421
Method: MLSTCA
y = 15.8 + 0.355 x R² = 0.094
ST: 1421
Method: cgMLSTCA
y = 12.5 + 0.436 x R² = 0.16
ST: 1421
Method: cgMLSTCRCA
y = 7.43 + 1.24 x R² = 0.51
ST: 1421
Method: SKA
y = 3.81 + 1.04 x R² = 0.8
ST: 1424
Method: MLSTCA
y = 2.08 + 0.859 x R² = 0.24
ST: 1424
Method: cgMLSTCA
y = 2.76 + 0.713 x R² = 0.15
ST: 1424
Method: cgMLSTCRCA
y = 7.71 + 0.927 x R² = 0.26
ST: 1424
Method: SKA
y = 1.88 + 1.36 x R² = 0.8
Pairwise distance using PCDR
Pairwise Distance
0 10 20 30 40 50

Supplementary Figure 4: Relationship between isolate-to-isolate pairwise distances.

Relationship between isolate-to-isolate pairwise distances between various genetic comparison methods (x-axis) and the pairwise comparison using *de novo* references (PCDR, y-axis) for each of the four major *E. faecium* STs. The pairwise distances shown on the x-axis are calculated from each of: the core alignments for each ST using a within-ST reference (MLSTCA), core alignments for each cgMLST cluster using a within-ST reference (cgMLSTCA), core alignments for each cgMLST cluster using a within-cgMLST cluster reference (cgMLSTCRCA) and split K-mer analysis (SKA). Data points are coloured based on method used. The blue line represents the line of best fit using a linear regression model (y ~ x) with the with the 95% confidence interval around the smoothing function shown in grey. Panel A shows all isolate pairs from a given ST. Panel B shows a subset of the same data as Panel A; x and y axis limits have been restricted to a maximum pairwise distance of $\leqslant$50 SNPs.

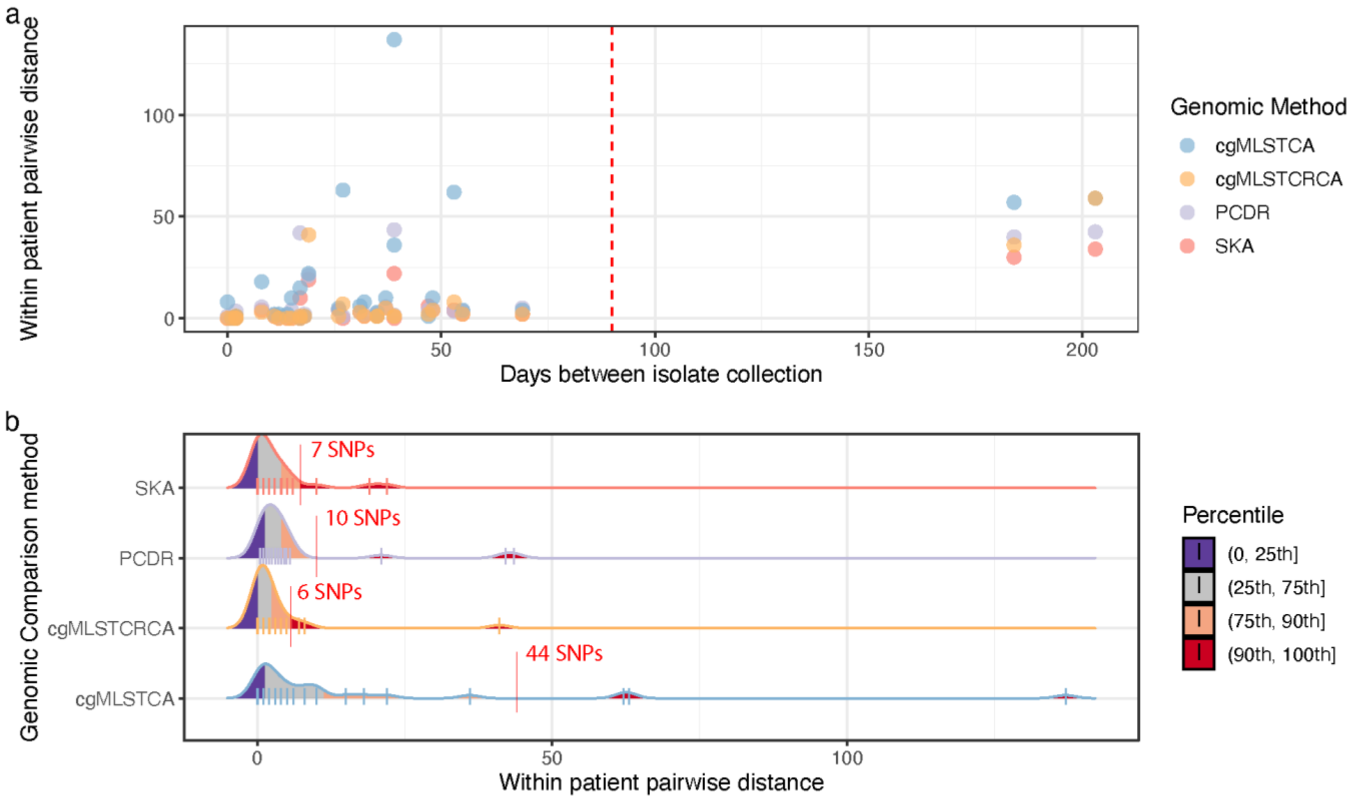

Supplementary Figure 5: Intrapatient isolate diversity. a) The number of days between isolate collection for each intrapatient pair (n=30). The red dashed line indicates the 90-day threshold. b) Density distribution of the within patient isolate pairs with temporal-outliers removed (n=28). The 90th percentile SNP patient pairwise distance is displayed in red. Isolate pairs with a time between isolation of 90 days or more were removed from b).

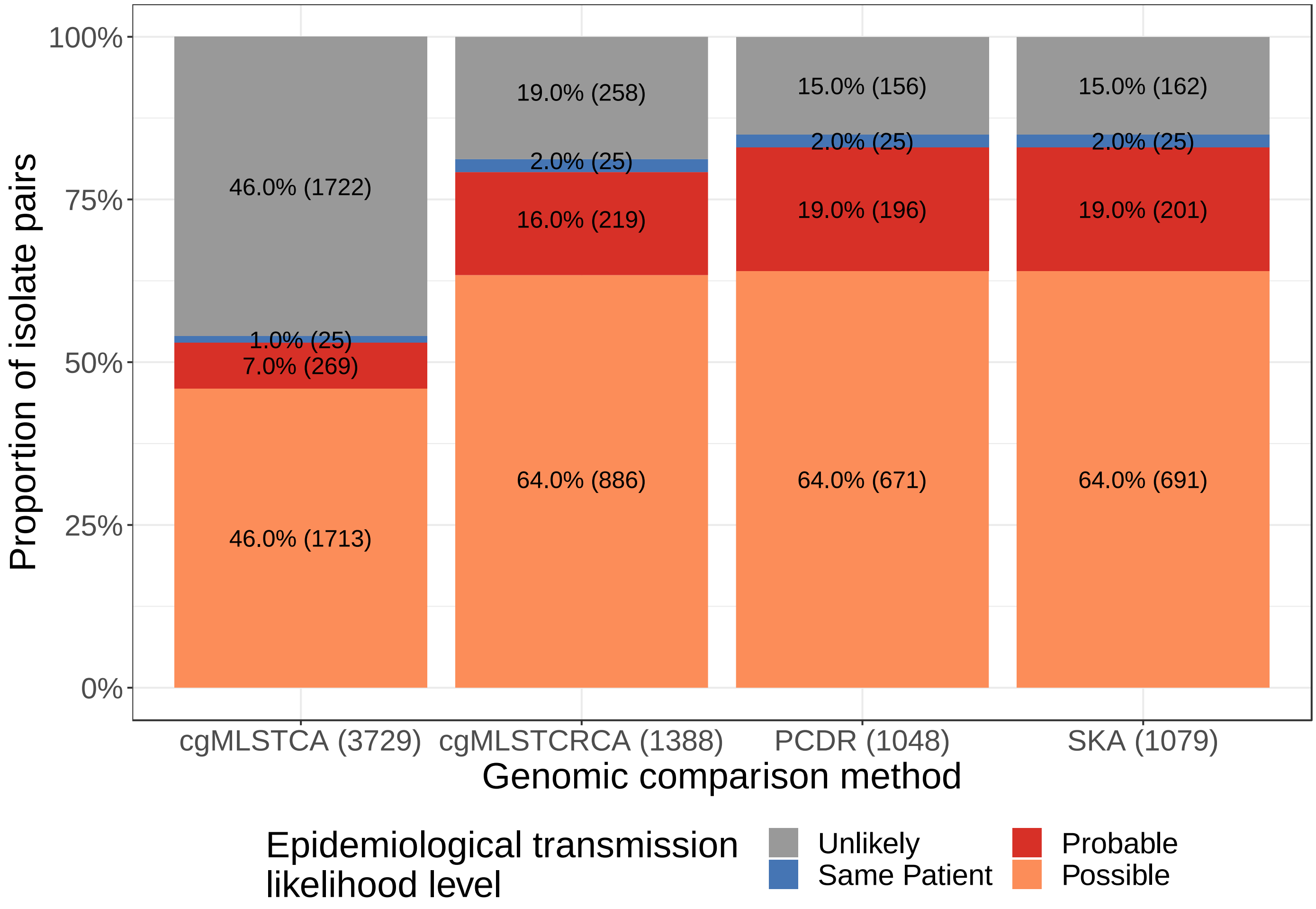

Supplementary Figure 6: Proportion of isolate pairs with pairwise SNP distances below the genomic threshold of putative transmission. Count of genomic transmission links at each epidemiological transmission likelihood level displayed in brackets. Includes only isolates pairs that are equal to or less than each method's genomic transmission link threshold as determined by intrapatient SNP diversity (cgMLSTCA: ⩽44 SNPs, cgMLSTCRCA: ⩽6 SNPs, PCDR: ⩽10 SNPs, SKA: ⩽7 SNPs).

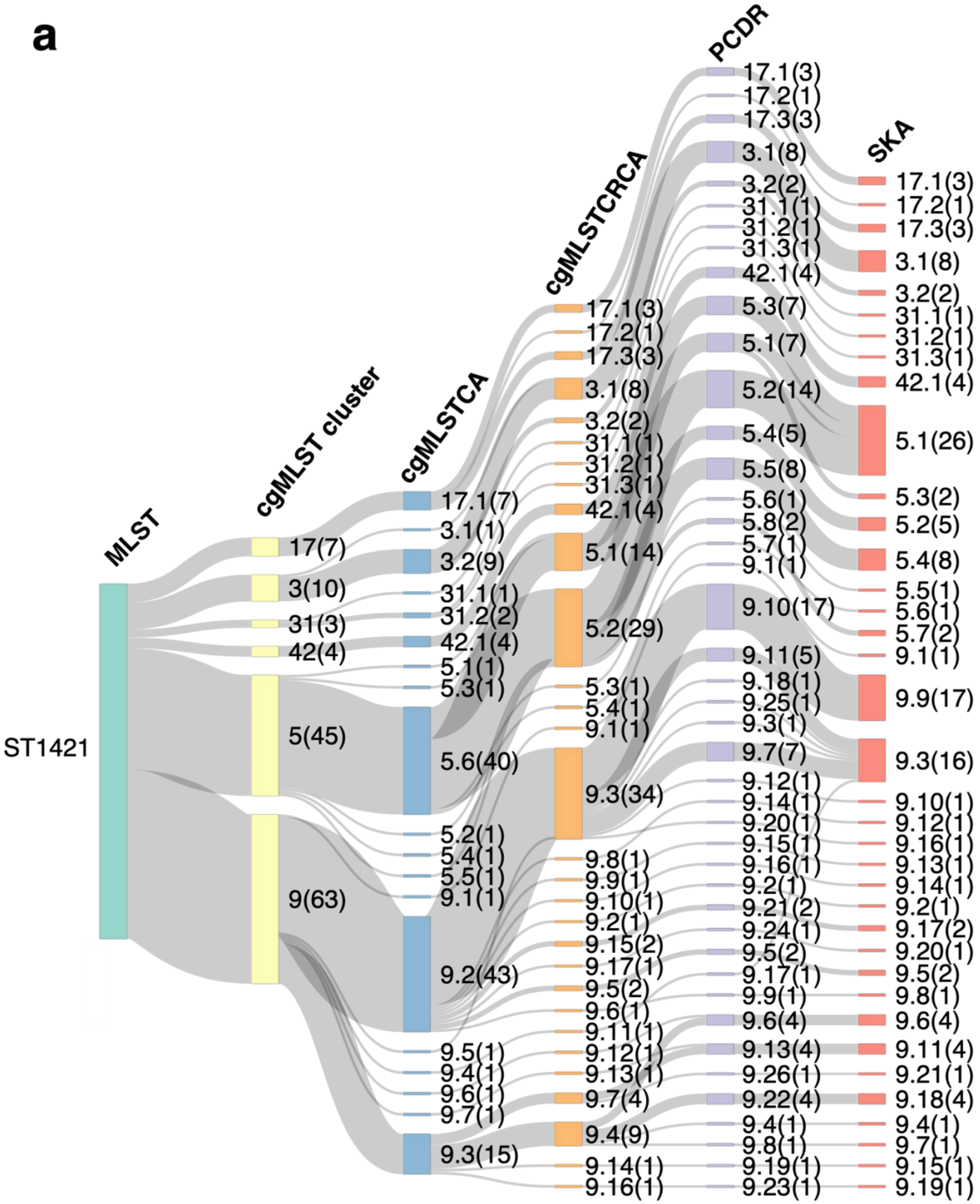
a
MLST
cgMLST cluster
cgMLSTCA
cgMLSTCRCA
PCDR
SKA
ST1421
17(7)
3(10)
31(3)
42(4)
5(45)
9(63)
17.1(7)
3.1(1)
3.2(9)
31.1(1)
31.2(2)
42.1(4)
5.1(1)
5.3(1)
5.6(40)
5.2(1)
5.4(1)
5.5(1)
9.1(1)
9.2(43)
9.5(1)
9.4(1)
9.6(1)
9.7(1)
9.3(15)
17.1(3)
17.2(1)
17.3(3)
3.1(8)
3.2(2)
31.1(1)
31.2(1)
31.3(1)
42.1(4)
5.1(14)
5.2(29)
5.3(1)
5.4(1)
9.1(1)
9.3(34)
9.8(1)
9.9(1)
9.10(1)
9.2(1)
9.15(2)
9.17(1)
9.5(2)
9.6(1)
9.11(1)
9.12(1)
9.13(1)
9.7(4)
9.4(9)
9.14(1)
9.16(1)
17.1(3)
17.2(1)
17.3(3)
3.1(8)
3.2(2)
31.1(1)
31.2(1)
31.3(1)
42.1(4)
5.3(7)
5.1(7)
5.2(14)
5.4(5)
5.5(8)
5.6(1)
5.8(2)
5.7(1)
9.1(1)
9.10(17)
9.11(5)
9.18(1)
9.25(1)
9.3(1)
9.7(7)
9.12(1)
9.14(1)
9.20(1)
9.15(1)
9.16(1)
9.2(1)
9.21(2)
9.24(1)
9.5(2)
9.17(1)
9.9(1)
9.6(4)
9.13(4)
9.26(1)
9.22(4)
9.4(1)
9.8(1)
9.19(1)
9.23(1)
17.1(3)
17.2(1)
17.3(3)
3.1(8)
3.2(2)
31.1(1)
31.2(1)
31.3(1)
42.1(4)
5.1(26)
5.3(2)
5.2(5)
5.4(8)
5.5(1)
5.6(1)
5.7(2)
9.1(1)
9.9(17)
9.3(16)
9.10(1)
9.12(1)
9.16(1)
9.13(1)
9.14(1)
9.2(1)
9.17(2)
9.20(1)
9.5(2)
9.8(1)
9.6(4)
9.11(4)
9.21(1)
9.18(4)
9.4(1)
9.7(1)
9.15(1)
9.19(1)

b

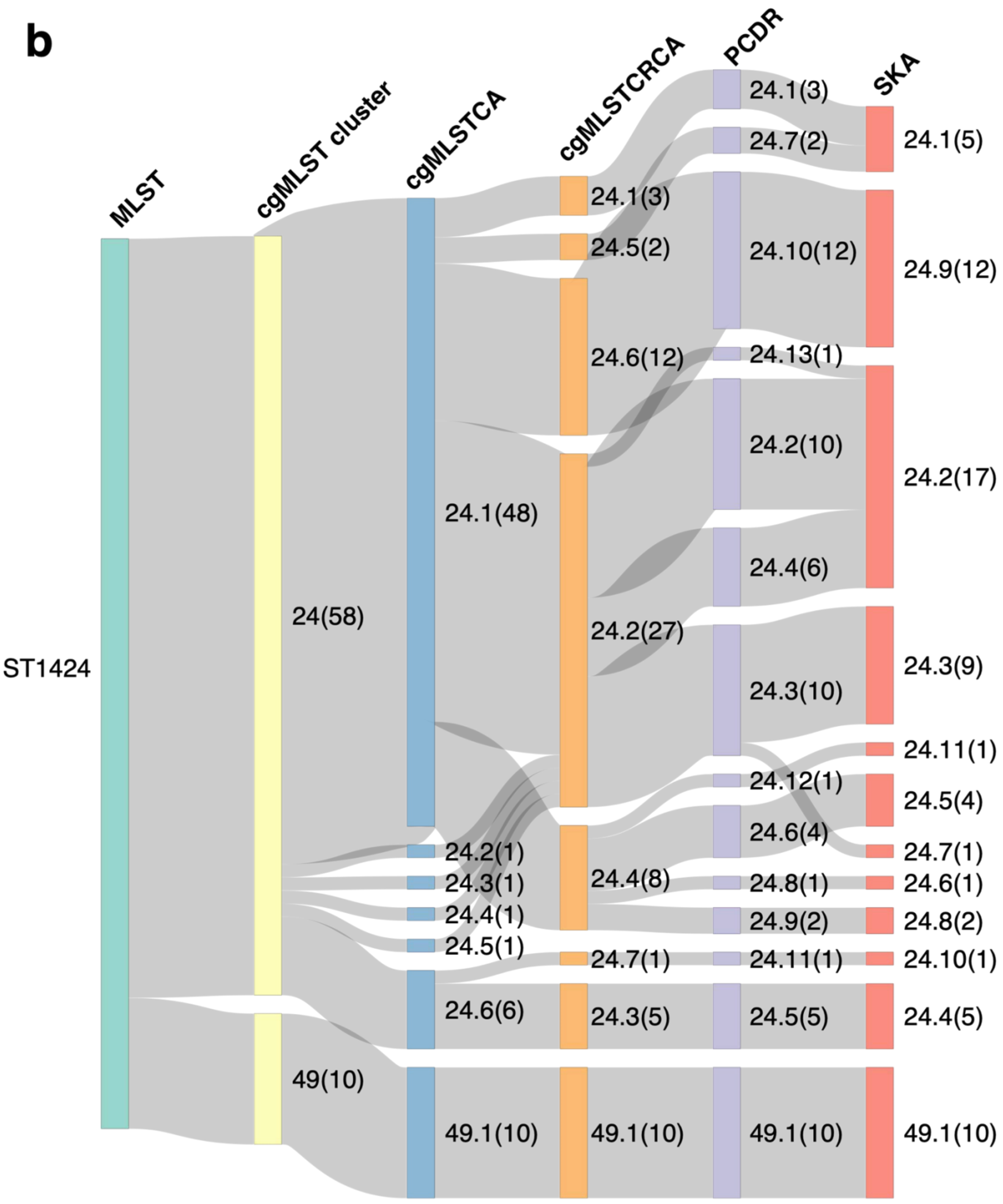

MLST
cgMLST cluster
cgMLSTCA
cgMLSTCRCA
PCDR
SKA
ST1424
24(58)
49(10)
24.1(48)
24.2(1)
24.3(1)
24.4(1)
24.5(1)
24.6(6)
49.1(10)
24.1(3)
24.5(2)
24.6(12)
24.2(27)
24.4(8)
24.7(1)
24.3(5)
49.1(10)
24.1(3)
24.7(2)
24.10(12)
24.13(1)
24.2(10)
24.4(6)
24.3(10)
24.12(1)
24.6(4)
24.8(1)
24.9(2)
24.11(1)
24.5(5)
49.1(10)
24.1(5)
24.9(12)
24.2(17)
24.3(9)
24.11(1)
24.5(4)
24.7(1)
24.6(1)
24.8(2)
24.10(1)
24.4(5)
49.1(10)

c

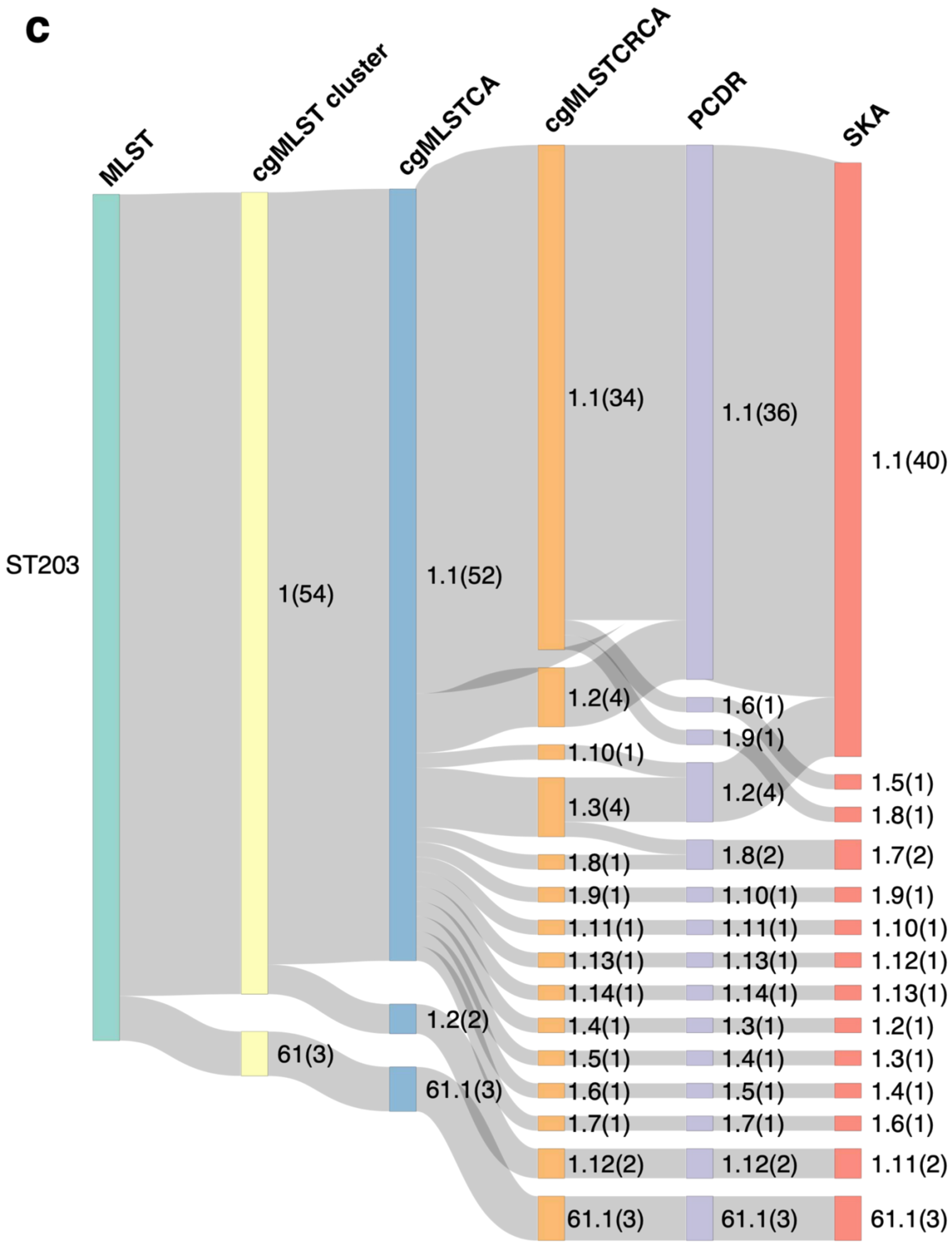
MLST
cgMLST cluster
cgMLSTCA
cgMLSTCRCA
PCDR
SKA
ST203
1(54)
61(3)
1.1(52)
1.2(2)
61.1(3)
1.1(34)
1.2(4)
1.10(1)
1.3(4)
1.8(1)
1.9(1)
1.11(1)
1.13(1)
1.14(1)
1.4(1)
1.5(1)
1.6(1)
1.7(1)
1.12(2)
61.1(3)
1.1(36)
1.6(1)
1.9(1)
1.2(4)
1.8(2)
1.10(1)
1.11(1)
1.13(1)
1.14(1)
1.3(1)
1.4(1)
1.5(1)
1.7(1)
1.12(2)
61.1(3)
1.1(40)
1.5(1)
1.8(1)
1.7(2)
1.9(1)
1.10(1)
1.12(1)
1.13(1)
1.2(1)
1.3(1)
1.4(1)
1.6(1)
1.11(2)
61.1(3)

d

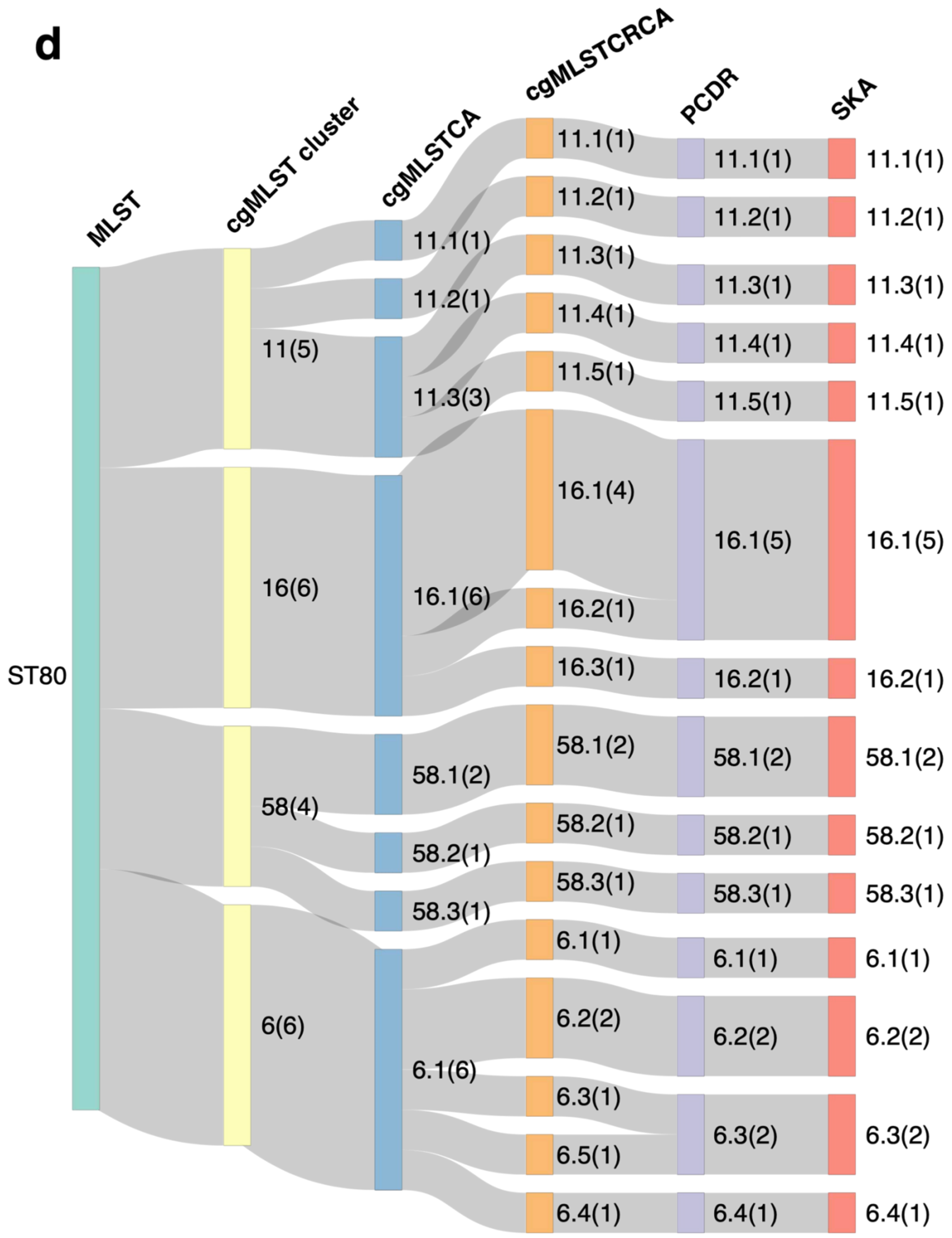
MLST
cgMLST cluster
cgMLSTCA
cgMLSTCRCA
PCDR
SKA
ST80
11(5)
16(6)
58(4)
6(6)
11.1(1)
11.2(1)
11.3(3)
16.1(6)
58.1(2)
58.2(1)
58.3(1)
6.1(6)
11.1(1)
11.2(1)
11.3(1)
11.4(1)
11.5(1)
16.1(4)
16.2(1)
16.3(1)
58.1(2)
58.2(1)
58.3(1)
6.1(1)
6.2(2)
6.3(1)
6.5(1)
6.4(1)
11.1(1)
11.2(1)
11.3(1)
11.4(1)
11.5(1)
16.1(5)
16.2(1)
58.1(2)
58.2(1)
58.3(1)
6.1(1)
6.2(2)
6.3(2)
6.4(1)
11.1(1)
11.2(1)
11.3(1)
11.4(1)
11.5(1)
16.1(5)
16.2(1)
58.1(2)
58.2(1)
58.3(1)
6.1(1)
6.2(2)
6.3(2)
6.4(1)

Supplementary Figure 7: Relationship between MLST, cgMLST, cgMLSTCA, cgMLSTCRCA, PCDR and SKA clusters for *E. faecium* isolates from the 4 major MLSTs (n=264). Panel a shows data for ST1421, panel b shows ST1424, panel c shows ST203 and panel d shows ST80. cgMLST clusters are based on single-linkage clustering using a pairwise allelic difference threshold of ⩽25 alleles. cgMLSTCA, cgMLSTCRCA, PCDR and SKA clusters are based on a pairwise SNP distance threshold determined based on intrapatient SNP diversity (cgMLSTCA: ⩽44 SNPs, cgMLSTCRCA: ⩽6 SNPs, PCDR: ⩽10 SNPs, SKA: <7 SNPs) and are generated using single linkage clustering. The size of the nodes represents the number of isolates in each of the clusters and is relative for each MLST and the number of isolates in each cluster is displayed in brackets.

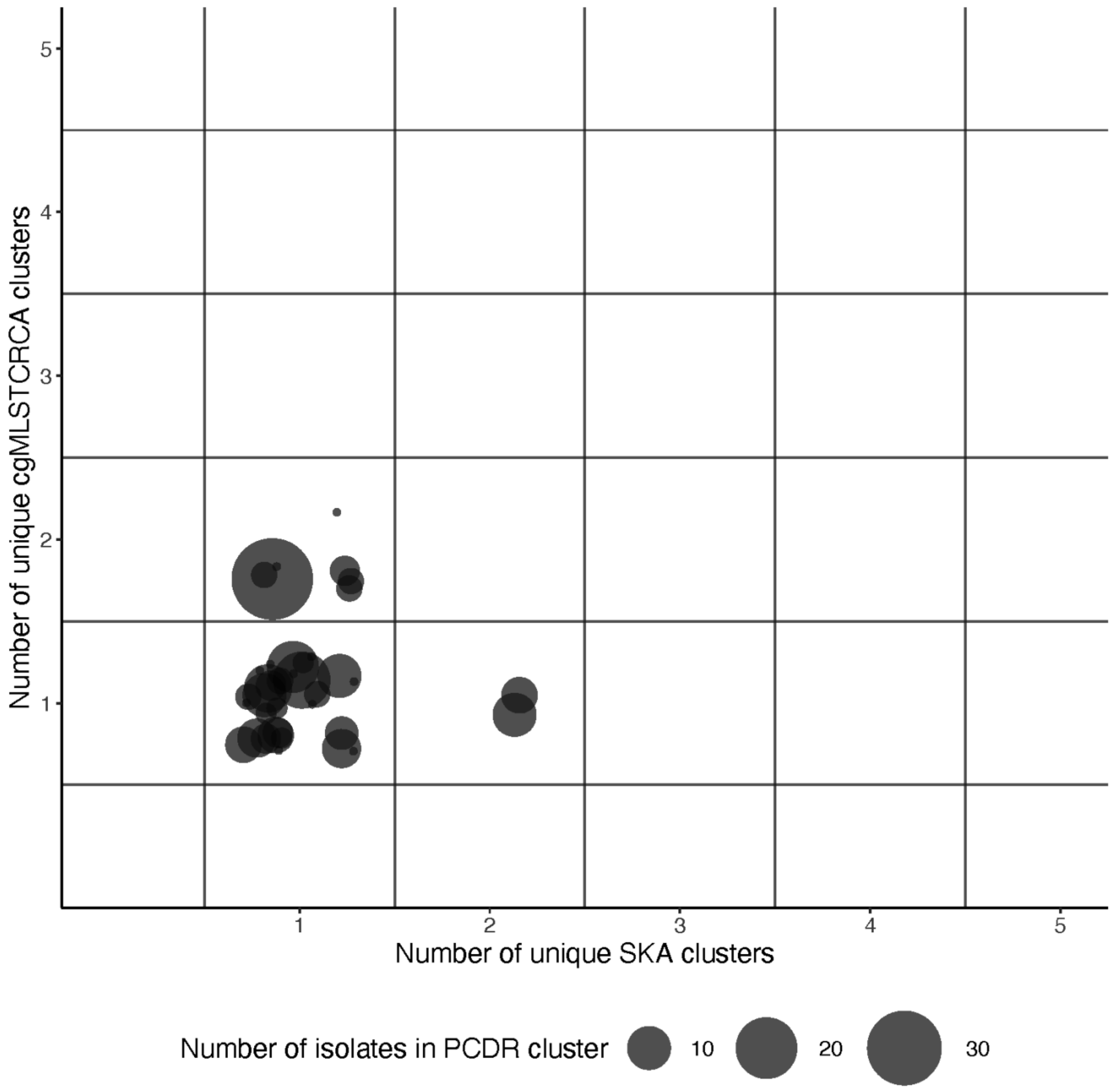

Supplementary Figure 8: The number of clusters each PCDR transmission cluster is divided into when using different genomic approaches. Only PCDR clusters with more than one isolate are shown (n=38). The total number of isolates in each cgMLST cluster is listed in brackets.

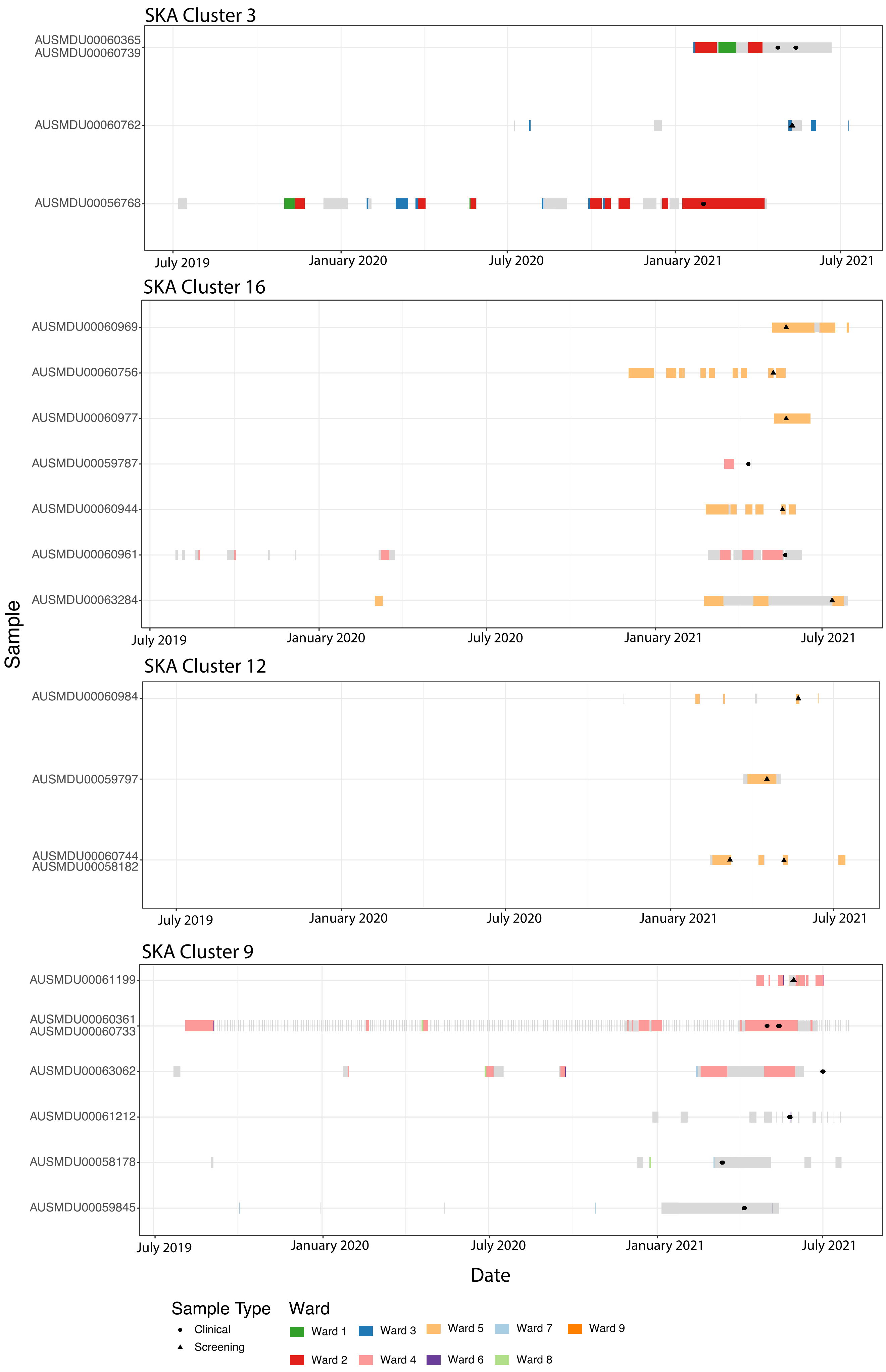

SKA Cluster 3
AUSMDU00060365
AUSMDU00060739
AUSMDU00060762
AUSMDU00056768
SKA Cluster 16
AUSMDU00060969
AUSMDU00060756
AUSMDU00060977
AUSMDU00059787
AUSMDU00060944
AUSMDU00060961
AUSMDU00063284
SKA Cluster 12
AUSMDU00060984
AUSMDU00059797
AUSMDU00060744
AUSMDU00058182
SKA Cluster 9
AUSMDU00061199
AUSMDU00060361
AUSMDU00060733
AUSMDU00063062
AUSMDU00061212
AUSMDU00058178
AUSMDU00059845
July 2019
January 2020
July 2020
January 2021
July 2021
Sample
Date
Sample Type
Clinical
Screening
Ward
Ward 1
Ward 2
Ward 3
Ward 4
Ward 5
Ward 6
Ward 7
Ward 8
Ward 9

Supplementary Figure 9: Gant chart of patient ward stays for the 4 major genomic transmission clusters. Samples were collected between January and June 2021 with the black shapes indicating the sample dates for the isolates. 2 years of patient move data was recorded (July 2019 to July 2021). Wards that were visited by only one patient within a genomic cluster have been greyed out as these are assumed to not be involved in transmission. Only genomic clusters containing four or more isolates have been plotted.

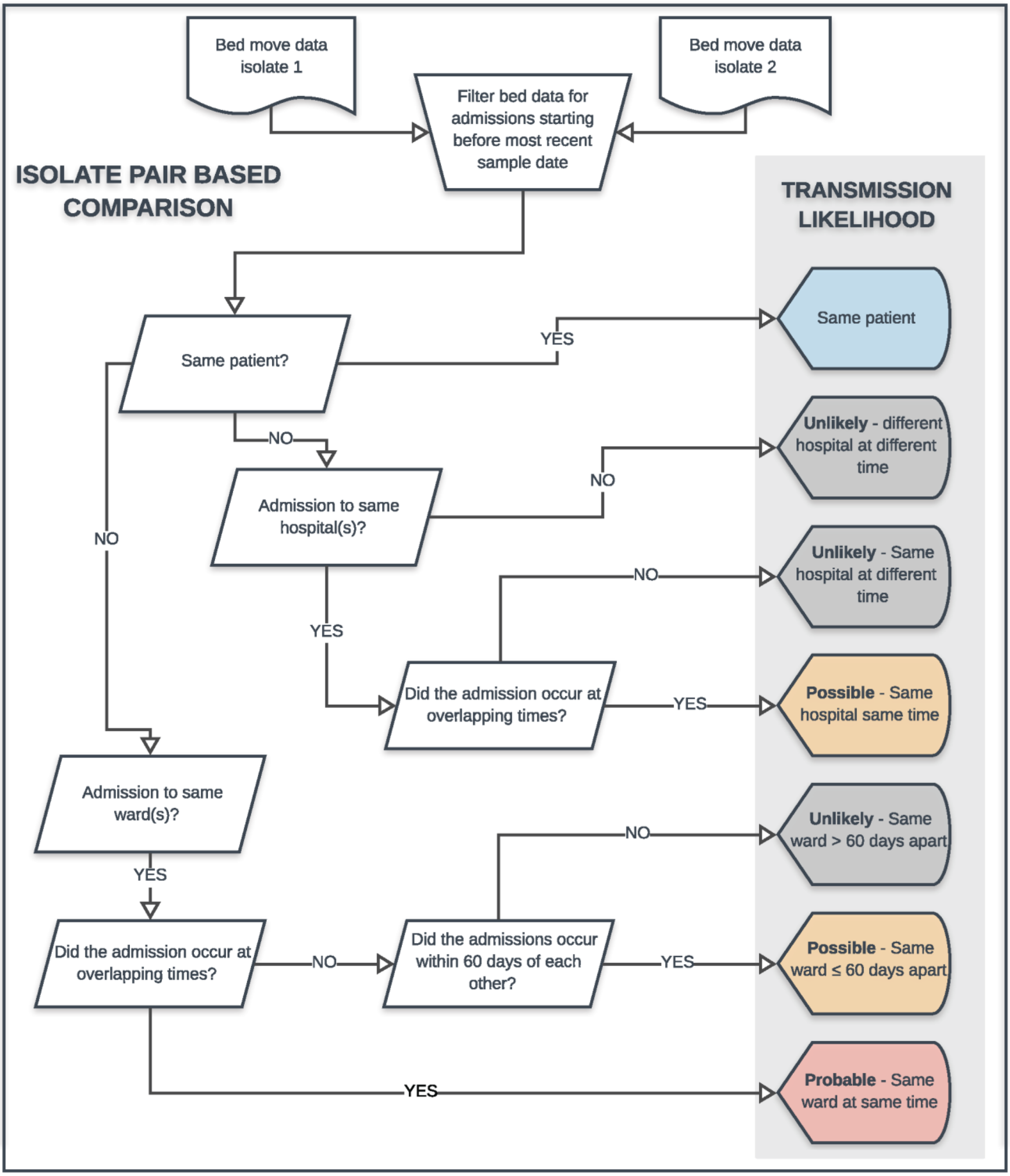

Supplementary Figure 10: Decision tree used to classify likelihood of transmission from epidemiologic data. Outlined is the decision algorithm used to classify the extent of epidemiological relatedness between each isolate pair.

a

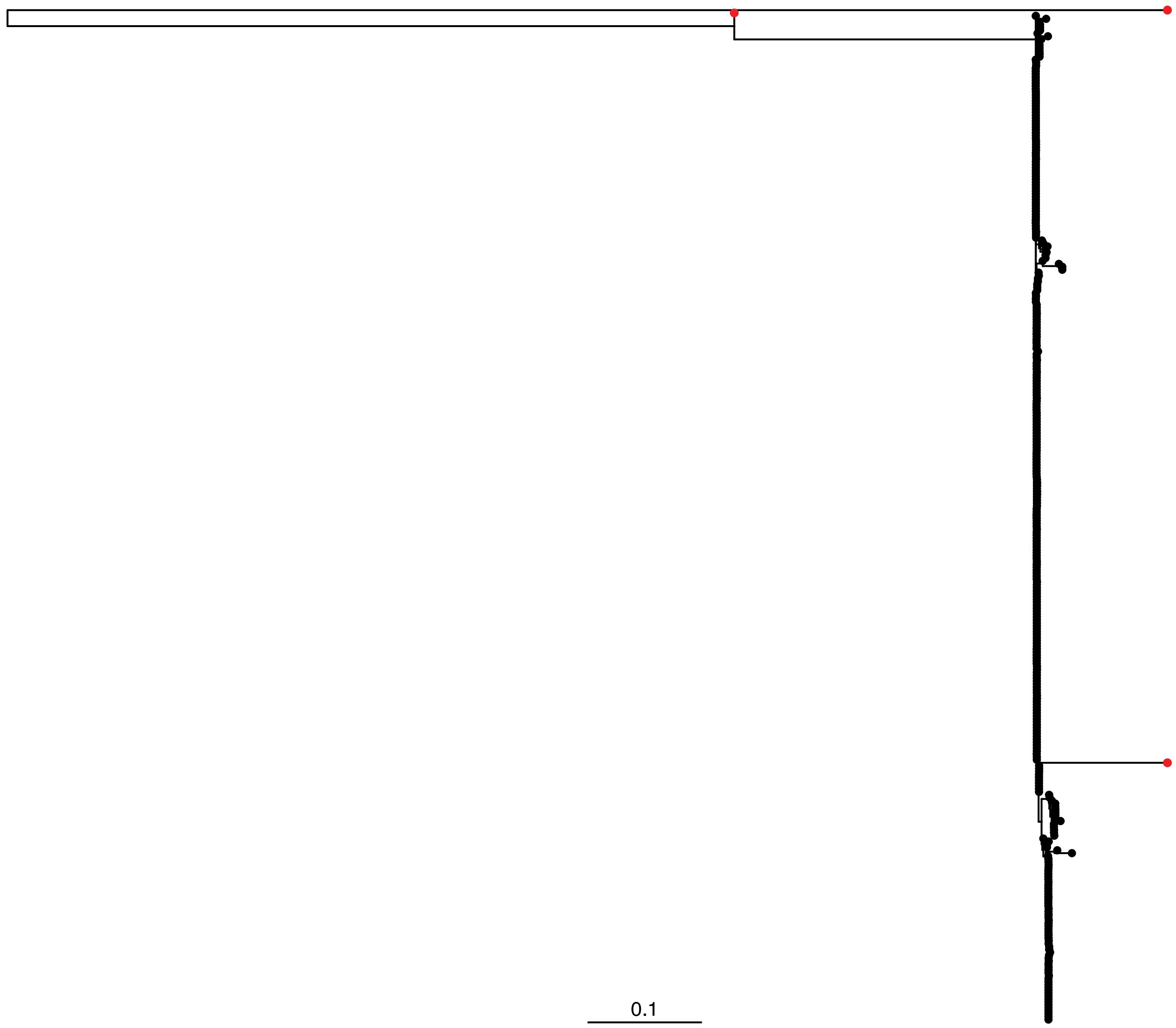

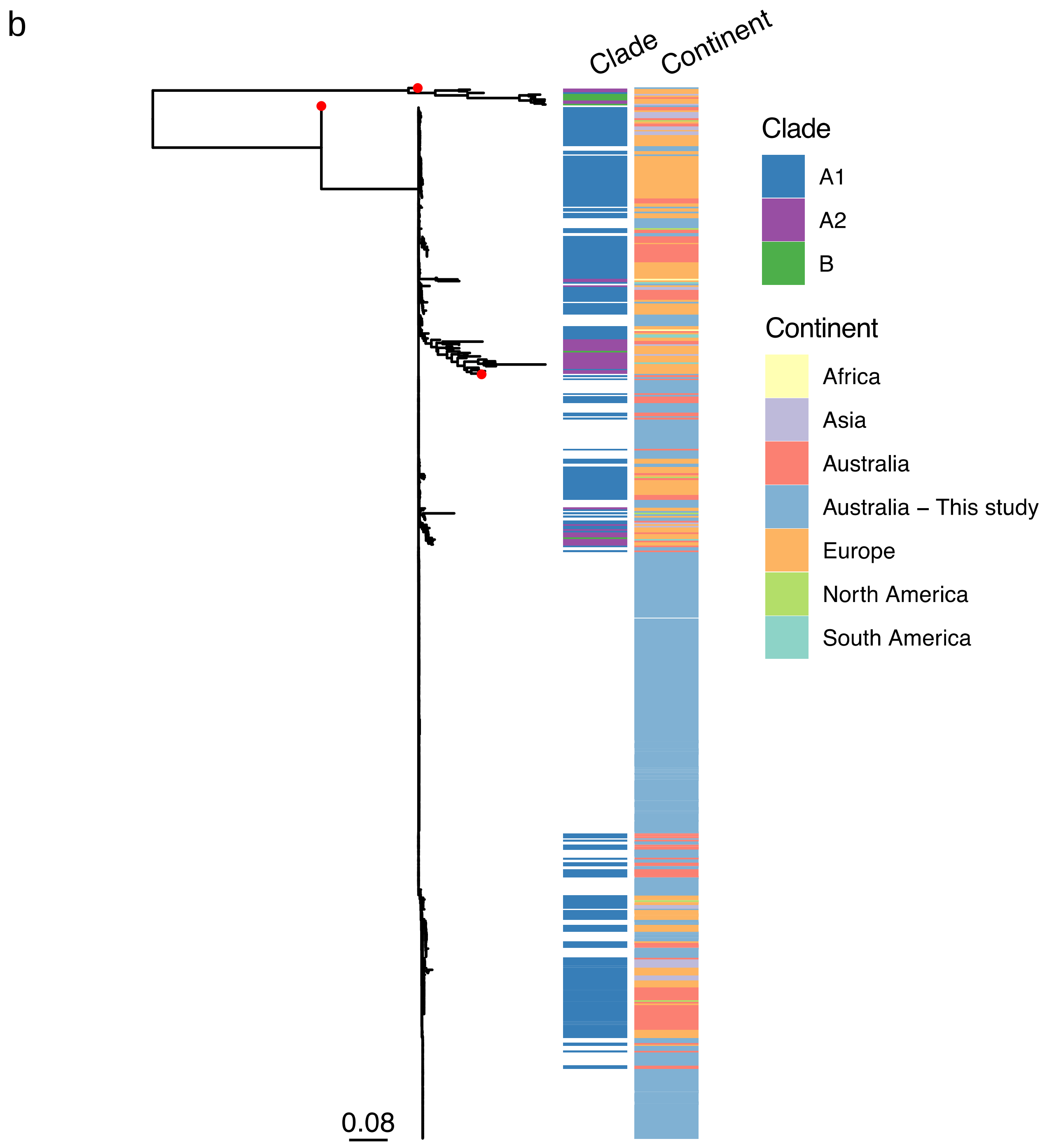

Supplementary Figure11: a) Midpoint-rooted maximum-likelihood phylogenetic tree of all *E. faecium* isolates. Tree includes all isolates used in this study (n=346). Outliers excluded from the main figure phylogenetic tree are identified in red. This tree was made using an alignment that was not masked for recombination. **b) Midpoint-rooted maximum likelihood phylogenetic tree of all study isolates and international context isolates.** Tree includes all isolates used in this study (n=346) and global context from van Hal et al. (1) (n=297). Study outliers excluded from the main figure phylogenetic tree are identified in red. This tree was made using an alignment that was not masked for recombination.

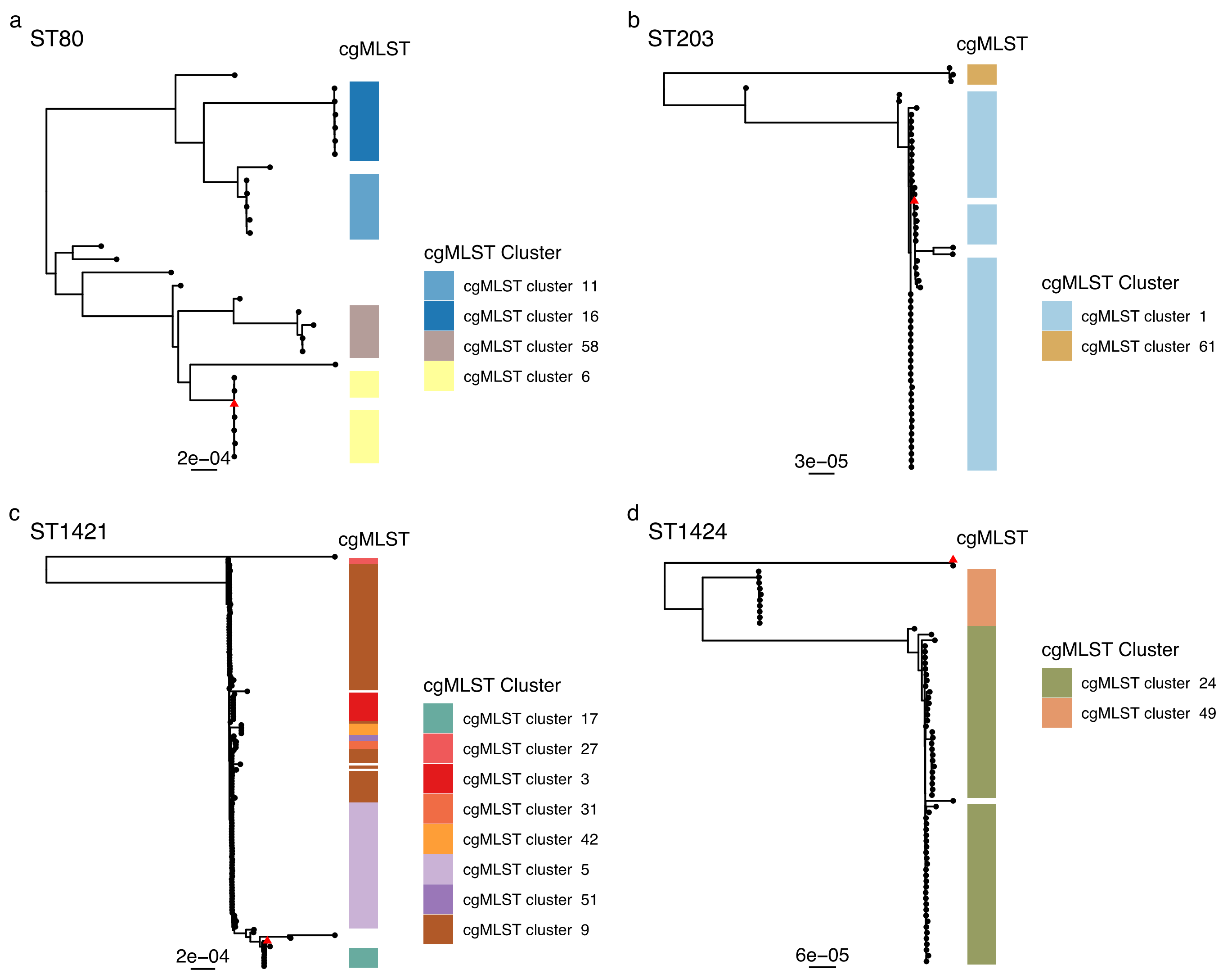

Supplementary Figure 12: Midpoint-rooted maximum-likelihood phylogenetic tree of *E. faecium* isolates. The reference genome is identified by the red triangle tip. Each tree represents isolates from one of the four major STs. Panel a shows ST80, panel b shows ST203, panel c shows ST1424 and panel d shows ST1421. cgMLST clusters with only one isolate are shown in white. All trees were made using alignments that did not have recombination masked.

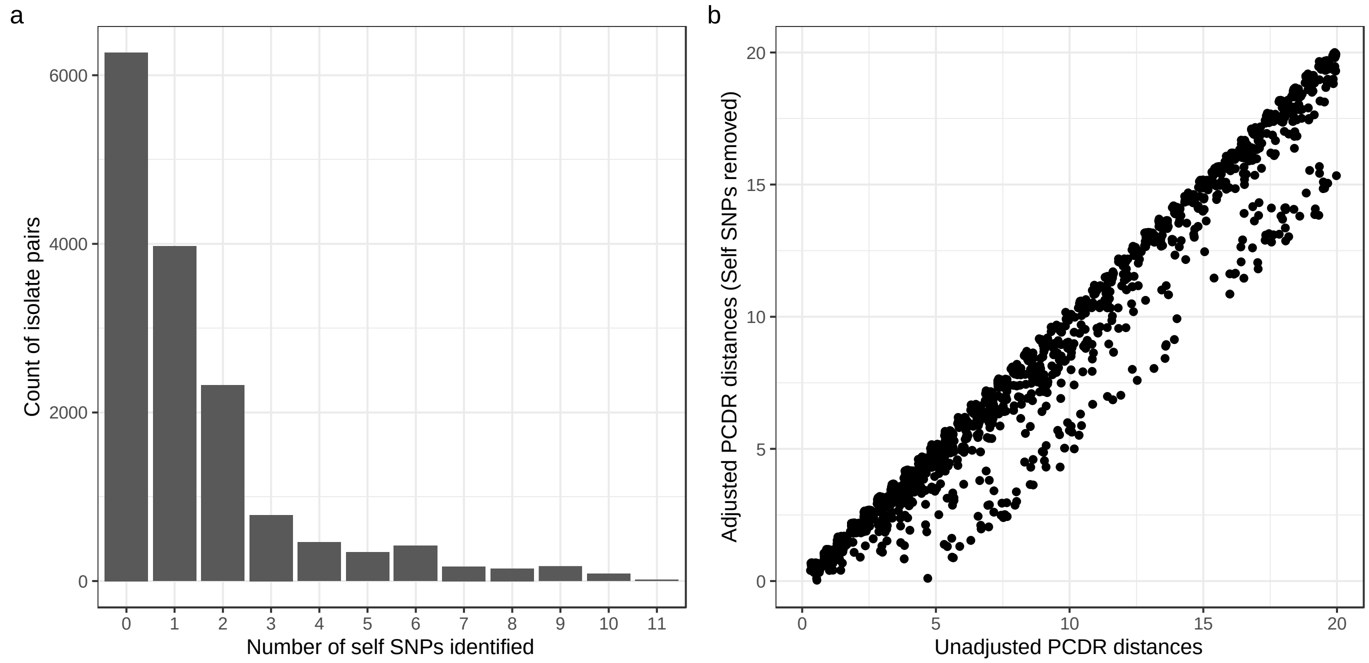

Supplementary Figure 13: Number of self SNPs identified and the corresponding adjusted PCDR distances. a) The number of self SNPs identified in SKESA assemblies. b) The adjusted and unadjusted PCDR SNP distances. Only closely related isolate pairs shown with unadjusted PCDR distances limited to less than 20 SNPs.

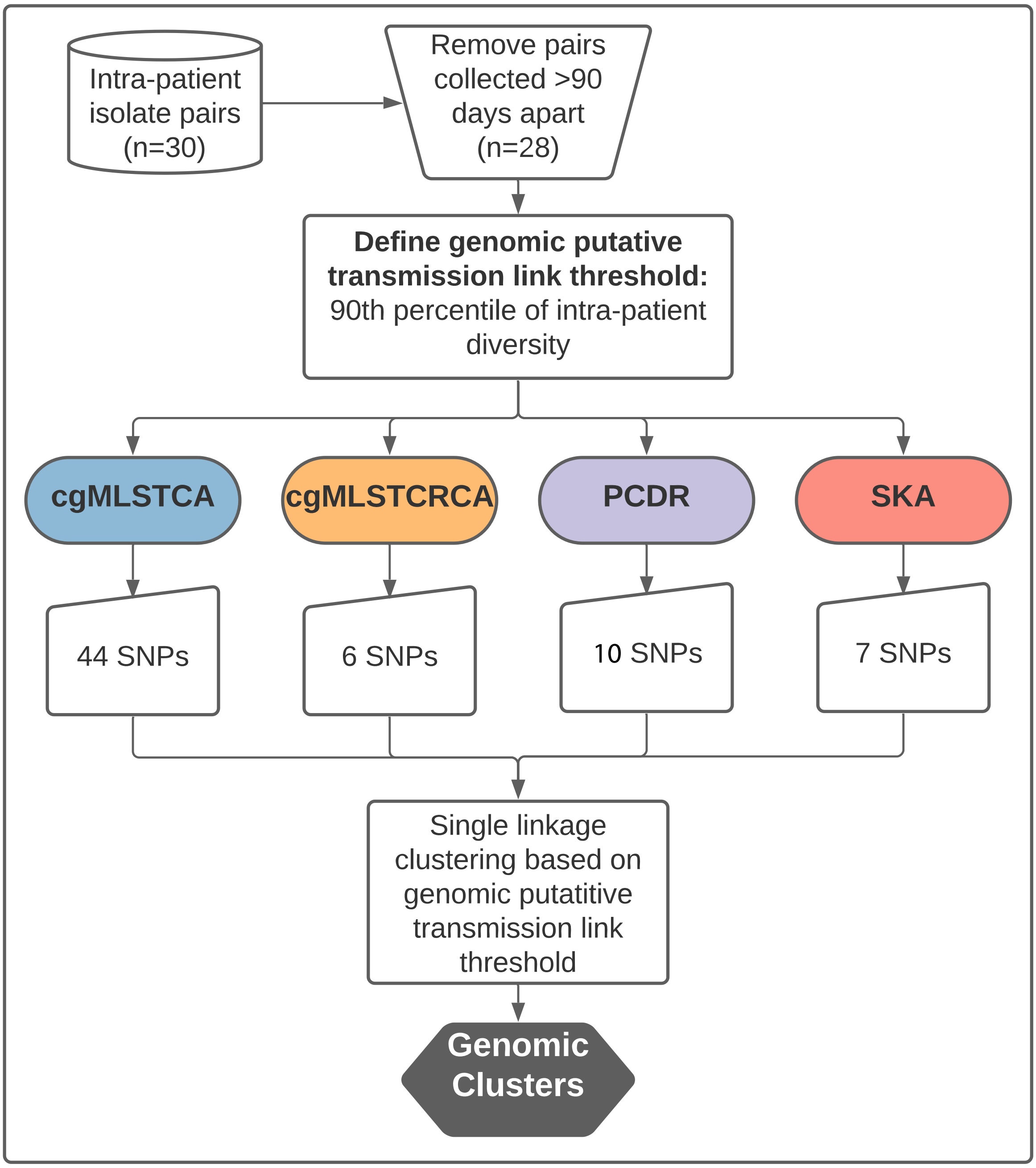

Supplementary Figure 14: Methods used to determine genomic transmission clusters. This flow chart outlines the methods that were used to determine the intrapatient diversity that would later be used as the threshold to determine the genomic putative transmission link threshold.

1. van Hal SJ, Willems RJL, Gouliouris T, Ballard SA, Coque TM, Hammerum AM, et al. The global dissemination of hospital clones of Enterococcus faecium. Genome Med. 2021 Mar 30;13(1):52.
