# Supplementary material for: Optimising genomic approaches for identifying vancomycin-resistant Enterococcus faecium transmission in healthcare settings
Source: Nat Commun. 2022 Jan 26;13:509. doi: 10.1038/s41467-022-28156-4 (PMC8792028; doi:10.1038/s41467-022-28156-4)
Supplement: Supplementary file 3 — Description of Additional Supplementary Files [file 41467_2022_28156_MOESM3_ESM.pdf]

Description of Additional Supplementary Files

Supplementary Data 1: List of accession numbers for all isolates included in the study and associated metadata.

Supplementary Data 2: List of all reference genomes used in the study and summary statistics for all the core genome alignments.

Supplementary Data 3: Pairwise distance data for all methods used in the study, including patient ward move data.

Supplementary Data 4: Isolate list and metadata from the hospital case study, including patient ward move data.

Supplementary Data 5: Code used to generate the genomic comparison data.
