# Supplementary material for: Optimising genomic approaches for identifying vancomycin-resistant Enterococcus faecium transmission in healthcare settings
Source: Nat Commun. 2022 Jan 26;13:509. doi: 10.1038/s41467-022-28156-4 (PMC8792028; doi:10.1038/s41467-022-28156-4)
Supplement: Supplementary file 9 — Reporting Summary [file 41467_2022_28156_MOESM9_ESM.pdf]

nature research

Corresponding author(s): Benjamin Howden

Last updated by author(s): Dec 5, 2021

Reporting Summary

Nature Research wishes to improve the reproducibility of the work that we publish. This form provides structure for consistency and transparency in reporting. For further information on Nature Research policies, see our Editorial Policies and the Editorial Policy Checklist.

Statistics

For all statistical analyses, confirm that the following items are present in the figure legend, table legend, main text, or Methods section.

n/a	Confirmed	
[ ]	[x]	The exact sample size (n) for each experimental group/condition, given as a discrete number and unit of measurement
[ ]	[x]	A statement on whether measurements were taken from distinct samples or whether the same sample was measured repeatedly
[x]	[ ]	The statistical test(s) used AND whether they are one- or two-sided<br>*Only common tests should be described solely by name; describe more complex techniques in the Methods section.*
[ ]	[x]	A description of all covariates tested
[x]	[ ]	A description of any assumptions or corrections, such as tests of normality and adjustment for multiple comparisons
[ ]	[x]	A full description of the statistical parameters including central tendency (e.g. means) or other basic estimates (e.g. regression coefficient) AND variation (e.g. standard deviation) or associated estimates of uncertainty (e.g. confidence intervals)
[x]	[ ]	For null hypothesis testing, the test statistic (e.g. F, t, r) with confidence intervals, effect sizes, degrees of freedom and P value noted<br>*Give P values as exact values whenever suitable.*
[x]	[ ]	For Bayesian analysis, information on the choice of priors and Markov chain Monte Carlo settings
[x]	[ ]	For hierarchical and complex designs, identification of the appropriate level for tests and full reporting of outcomes
[x]	[ ]	Estimates of effect sizes (e.g. Cohen's d, Pearson's r), indicating how they were calculated

Our web collection on statistics for biologists contains articles on many of the points above.

Software and code

Policy information about availability of computer code

Data collection	No software was used for data collection.
Data analysis	See Methods for settings used for all tools<br>mlst v2.19.0 (https://github.com/tseemann/mlst)<br>chewBACCA v2.0.16 (https://github.com/B-UMMI/chewBBACA)<br>COREugate v2.0.4 (https://github.com/MDU-PHL/Coreugate)<br>porechop v0.2.4 (https://github.com/rrwick/Porechop)<br>filtlong v0.2.0 (https://github.com/rrwick/Filtlong)<br>trycycler v0.3.3 (https://github.com/rrwick/Trycycler)<br>flye v2.8.1 https://github.com/fenderglass/Flye)<br>miniasm+minipolish v.2.17 (https://github.com/lh3/miniasm)<br>raven V1.1.0 (https://github.com/lbcb-sci/raven)<br>snippy v4.6.0 (https://github.com/tseemann/snippy)<br>bcftools v1.9 (https://github.com/samtools/bcftools)<br>IQtree v1.6.12 (https://github.com/Cibiv/IQ-TREE)<br>Gubbins v2.4.1 (https://github.com/sanger-pathogens/gubbins)<br>SKESA v2.3.0 (https://github.com/ncbi/SKESA)<br>SKA v1.0 (https://github.com/simonrharris/SKA)<br>R v4.0.2 (https://www.r-project.org/)<br>phangorn v2.5.5 (https://github.com/KlausVigo/phangorn)<br>ape v5.4 (https://cran.r-project.org/web/packages/ape/index.html)<br>ggtree v2.3.4 (https://github.com/YuLab-SMU/ggtree)

ggplot v3.3.2 (https://github.com/tidyverse/ggplot2)
harrietr v0.2.3 (https://github.com/andersgs/harrietr)
tidyverse v1.3.0 (https://github.com/tidyverse/tidyverse)
networkD3 v0.4 (https://github.com/christophergandrud/networkD3)
naniar v0.5.2 (https://github.com/njtierney/naniar)
ggpubr v0.4.0 (https://github.com/kassambara/ggpubr)
patchwork v1.0.1 (https://github.com/thomasp85/patchwork)
htmlwidgets v1.5.3 (https://github.com/ramnathv/htmlwidgets)
htmltools v0.5.1.1 (https://github.com/rstudio/htmltools)
ggpmisc v0.3.6 (https://github.com/cran/ggpmisc)

For manuscripts utilizing custom algorithms or software that are central to the research but not yet described in published literature, software must be made available to editors and reviewers. We strongly encourage code deposition in a community repository (e.g. GitHub). See the Nature Research guidelines for submitting code & software for further information.

Data

Policy information about availability of data

All manuscripts must include a data availability statement. This statement should provide the following information, where applicable:

- Accession codes, unique identifiers, or web links for publicly available datasets
- A list of figures that have associated raw data
- A description of any restrictions on data availability

Illumina sequencing reads for all samples from the formal “Controlling Superbugs” were deposited into GenBank under BioProject PRJNA565795 [https://www.ncbi.nlm.nih.gov/bioproject/PRJNA565795/]. Illumina sequence reads for all samples part of the case study were deposited into BioProject PRJEB49226 [https://www.ncbi.nlm.nih.gov/bioproject/ PRJEB49226/]. Reference assemblies used can be found in BioProject PRJNA565795 [https://www.ncbi.nlm.nih.gov/bioproject/PRJNA565795/] or PRJNA433676 [https://www.ncbi.nlm.nih.gov/bioproject/PRJNA433676/]. A full isolate list and associated metadata can be found in Supplementary Data 1. Supplementary Data 2 contains a list of all reference genomes used in the study and summary statistics for the core genome alignments. Genetic distance data for the method comparison and ward move data can be found in Supplementary Data 3. An isolate list, associated metadata and ward move data for the hospital case study can be found in Supplementary Data 4. Only the processed ward move data is available in the supplementary data, the raw ward move data are not available due to data privacy laws.

Field-specific reporting

Please select the one below that is the best fit for your research. If you are not sure, read the appropriate sections before making your selection.

☒ Life sciences ☐ Behavioural & social sciences ☐ Ecological, evolutionary & environmental sciences

For a reference copy of the document with all sections, see nature.com/documents/nr-reporting-summary-flat.pdf

Life sciences study design

All studies must disclose on these points even when the disclosure is negative.

Sample size	Sample size was determined based on the number of vancomycin resistant enterococcus faecium (VREfm) samples that were collected as part of the Controlling Superbugs study (n=346). These isolates were collected over a total of 15 months across 4 hospital networks in Victoria, Australia. In addition, 50 isolates (collected over a 6 month period) were used in the single hospital case study. Although no explicit sample size calculation was performed, these sample sizes are sufficient as the samples were collected over a time period and geography that ensured both a large diversity of samples was collected and that multiple transmission events could be captured.
Data exclusions	To ensure high quality analysis, we applied quality control criteria for the sequence data. All VREfm samples collected as part of the Controlling Superbugs study were included in the analysis.
Replication	Genomic analysis techniques were applied to individual multi locus sequence type (MLST) and core genome MLST (cgMLST) groups/clusters in order to verify the applicability of results across multiple genetic backgrounds.
Randomization	Randomisation was not relevant to this study as all samples were subjected to the same genomic analyses in order to fully characterise the dataset.
Blinding	Scientists performing genomic analyses were blinded to the patient and epidemiologic data, such that genomic cluster determination was performed without knowledge of epidemiologic clustering.

Reporting for specific materials, systems and methods

We require information from authors about some types of materials, experimental systems and methods used in many studies. Here, indicate whether each material, system or method listed is relevant to your study. If you are not sure if a list item applies to your research, read the appropriate section before selecting a response.

Materials & experimental systems

n/a	Involved in the study
☒	☐ Antibodies
☒	☐ Eukaryotic cell lines
☒	☐ Palaeontology and archaeology
☒	☐ Animals and other organisms
☒	☐ Human research participants
☒	☐ Clinical data
☒	☐ Dual use research of concern

Methods

n/a	Involved in the study
☒	☐ ChIP-seq
☒	☐ Flow cytometry
☒	☐ MRI-based neuroimaging
